# Safety evaluation of root extract of *Pueraria lobata* and *Scutellaria baicalensis* in rats

**DOI:** 10.1186/s12906-020-02998-1

**Published:** 2020-07-17

**Authors:** Jungbin Song, Young-Sik Kim, Donghun Lee, Hocheol Kim

**Affiliations:** 1 Department of Herbal Pharmacology, College of Korean Medicine, Kyung Hee University, 26 Kyungheedae-ro, Dongdaemun-gu, Seoul, 02447 Republic of Korea; 2 Department of Herbal Pharmacology, College of Korean Medicine, Gachon University, 1342 Seongnamdae-ro, Sujeong-gu, Seongnam-si, Gyeonggi-do 13120 Republic of Korea

**Keywords:** *Pueraria lobata*, *Scutellaria baicalensis*, Safety, Approximate lethal dose, HT047

## Abstract

**Background:**

The roots of *Pueraria lobata* and *Scutellaria baicalensis*, herbal medicines with a long history of widespread use, have been traditionally prescribed in combination to treat stroke, diabetes, and acute infectious diarrhea in East Asia. Nevertheless, toxicological data on these herbs and their combination are limited. This study investigated the acute and 13-week subchronic toxicity of root extract of *P. lobata* and *S. baicalensis* (HT047) for stroke treatment in male and female Sprague-Dawley rats.

**Methods:**

In the acute toxicity study, HT047 was administered orally at a single dose of 5000 mg/kg. In the subchronic toxicity study, HT047 was administered orally at repeated daily doses of 800, 2000, and 5000 mg/kg/day for 13 weeks, followed by a 4-week recovery period.

**Results:**

In the acute toxicity study, there were no deaths or toxicologically significant changes in clinical signs, body weight, and necropsy findings. In the subchronic toxicity study, HT047 at all doses caused no death and no treatment-related adverse effects on food consumption; organ weight; ophthalmologic, urinalysis, and hematological parameters; and necropsy findings of both rat sexes. There were some treatment-related alterations in clinical signs, body weight, and serum biochemistry and histopathological parameters; however, these changes were not considered toxicologically significant because they were resolved during the recovery period or resulted from the pharmacological effects of *P. lobata* and *S. baicalensis*.

**Conclusions:**

The oral approximate lethal dose (the lowest dose that causes mortality) of HT047 was greater than 5000 mg/kg in male and female rats. The oral no-observed-adverse-effect level of HT047 was greater than 5000 mg/kg/day in rats of both sexes, and no target organs were identified. The present findings support the safety of an herbal extract of *P. lobata* and *S. baicalensis* as a therapeutic agent for stroke and further confirm the safety of the combined use of *P. lobata* and *S. baicalensis* in clinical practice.

## Background

Stroke is the leading cause of long-term disability and death worldwide, yet effective treatments remain limited. Thrombolysis with intravenous tissue plasminogen activator is the only U.S. FDA approved pharmacologic treatment but only 3–5% of patients receive this treatment due to the risk of hemorrhagic complications, particularly symptomatic intracranial hemorrhage [1]. The majority of stroke patients are elderly with multiple comorbid conditions and polypharmacy who are more vulnerable to adverse drug reactions [2]. More than half of the patients report adverse events in stroke clinical trials [3], and several trials have been stopped prematurely because of an unfavorable risk-benefit ratio [4–6]. Therefore, safety should be a priority for new drug development for stroke.

In East Asia including China and Korea, traditional medicine has been historically used to treat stroke, and is still widely used today. Especially, the roots of *Pueraria lobata* (Willd.) Ohwi and *Scutellaria baicalensis* Georgi have a long history of use in the treatment of stroke [7]. *P. lobata* is a perennial vine belonging to the family Leguminosae, and its roots rich in isoflavones such as puerarin, daidzin, and daidzein are widely consumed as foods and medicines [8]. *S. baicalensis*, also known as Chinese skullcap, is a flowering plant in the family Lamiaceae and its roots, which contains various flavonoids including baicalin, baicalein, and wogonin, is one of the most frequently used herbal medicines in China and Korea [9]. These two herbs have been traditionally prescribed in combination to treat stroke, diabetes, and acute infectious diarrhea [10–12]. Inspired by traditional knowledge, our group have developed HT047, an herbal extract consisting of the roots of *P. lobata* and *S. baicalensis*, for promoting neuroprotection and neurorestoration following ischemic stroke. In preclinical studies, acute treatment with HT047 reduced infarct volume after cerebral ischemia in rats and delayed treatment initiated more than 24 h after ischemia improved long-term sensorimotor recovery in rats (data not published). Based on these findings, we decided to conduct a clinical trial to evaluate the efficacy and safety of HT047 in ischemic stroke patients [13].

Although *P. lobata* and *S. baicalensis* have a long history of widespread use, limited information is available regarding their toxicity in humans and animals. A few studies on general toxicity of each of two herbs have been conducted in animals [14–17]. However, these studies are single dose acute toxicity studies, and the effects of chronic exposure to each herb and their reversibility have not been well studied. Thus, repeated dose toxicity data, such as no-observed-adverse-effect level (NOAEL), dose-response relationship, and target organs, all of which are essential information, remain unknown. Long-term toxicity studies of *P. lobata* and *S. baicalensis*, to our knowledge, have only been conducted in combination with other herbs [18–21] and in these cases the administration doses were not high enough to ensure the clinical safety of each herb and HT047. Also, in the subchronic toxicity study of Keyler et al. (2002) [18], the toxicity of an herbal mixture containing *P. lobata* was assessed only in male rats, not in female rats. Furthermore, there has been no attempt to investigate the potential toxicity of combination of *P. lobata* and *S. baicalensis*, despite the widespread combined use in clinical practice. There is a misconception that herbal medicines are safe as they are natural and have been used since a long time, but they can cause significant adverse effects [22]. Recently, a few studies have reported the potential hepatotoxic effects of *P. lobata* [23], and kidney and heart injuries induced by the major active compounds of *S. baicalensis* when administered at relatively high doses [24, 25]. Therefore, a systemic toxicology study of HT047 in animals was essential to ensure its safety as a therapeutic agent for ischemic stroke prior to progressing to a clinical research.

In the present study, acute and subchronic oral toxicity tests of HT047 were conducted using both male and female Sprague-Dawley rats to evaluate the general toxicity and to determine the approximate lethal dose (ALD, i.e. the lowest dose that causes mortality), NOAEL, target organs, and reversibility of toxicity. The toxicity tests were conducted in compliance with the test guidelines of the Korean Ministry of Food and Drug Safety (MFDS).

## Methods

### Preparation of test substance

HT047 is a 30% ethanol extract of the dried roots of *P. lobata* and *S. baicalensis*. HT047 was produced by Bioland Co., Ltd. (Osong, Korea), a Bulk Good Manufacturing Practice certified manufacturer. The dried roots of *P. lobata* and *S. baicalensis* were authenticated by Professor Hocheol Kim of Kyung Hee University and mixed together in an 8.0:1.1 weight ratio. Voucher specimens of the authenticated raw materials were deposited in the Herbarium of Kyung Hee University College of Korean Medicine (No. 13070301 and 13070302). The mixed roots were extracted twice with 10 times (v/w) the amount of 30% (v/v) ethanol in distilled water for 3 h at 70 °C. The liquid extract was filtered, concentrated under 60 °C, and vacuum-dried to yield a yellowish-brown powder. The dried extract was used for high-performance liquid chromatography (HPLC) analysis and toxicity tests. Voucher specimens of the plants were deposited in the Department of Herbal Pharmacology, College of Korean Medicine, Kyung Hee University (HP028 and HP012).

### HPLC analysis

Puerarin and baicalin contents in HT047 samples were measured using HPLC before the toxicity studies. HPLC analysis was performed using a Waters instrument (MA, USA) equipped with a Waters 1525 pump, Waters 2707 autosampler, and Waters 2998 photodiode array detector. The separation was performed using a SunFire™ C18 column (250 × 4.6 mm i.d., 5 μm particle diameter, Waters). The mobile phase consisted of 1% (v/v) phosphoric acid in distilled water (A) and acetonitrile (B). The gradient profile was as follows: 0–60–61–63–68 min, 5–50–70–5–5% solvent B. The injection volume was 10 μL and the flow rate was maintained at 1.0 mL/min. The detection was monitored at 254 nm. The analytical methods were validated for selectivity, linearity, accuracy, and precision.

### Animals

Six-week-old Sprague-Dawley rats (Orientbio Inc., Korea) were used for the acute and subchronic toxicity studies. Animals were allowed free access to food (2918C, Harlan Laboratories, Inc., USA) and water and acclimated to housing conditions for 1 week prior to the experiments. Animals were housed under the following controlled conditions: 22 ± 3 °C, 50 ± 20% humidity, and a 12 h light-dark cycle.

### Study design overview

All experiments were conducted at Biotoxtech Co., Ltd., Korea in compliance with good laboratory practices. The acute toxicity study was conducted in compliance with the test guidelines of the Korean MFDS (No. 2013–121). The subchronic toxicity study was conducted in compliance with the test guidelines of the Korean MFDS (No. 2013–121) and Organization for Economic Co-operation and Development (OECD TG 408). This study was approved by the animal experiment committee of Biotoxtech Co., Ltd., Korea (Approval No. 130495 and 140014) on the basis of the Animal Protection Act of Korea.

### Acute oral toxicity study

**Fig. 1:**
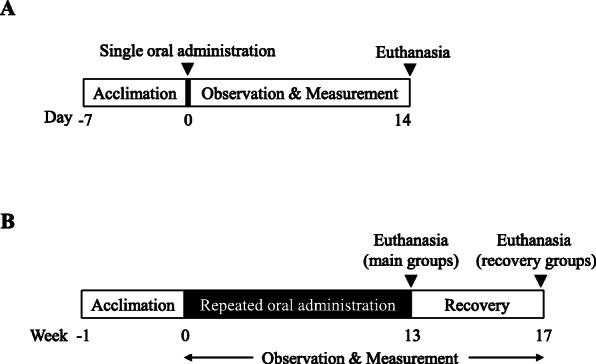
Experimental timelines (**a**) Acute oral toxicity study (**b**) subchronic toxicity study

A total of twenty 6-week-old Sprague-Dawley rats were randomly assigned to the control or HT047 groups (n = 10; 5 males and 5 females per group). HT047 was administered by oral gavage at a single dose of 5000 mg/kg in a volume of 10 mL/kg body weight. The control group received the vehicle (sterile water for injection; Choongwae Pharma Corp., Korea) in the same regimen. Clinical signs were observed for 14 days, and body weight was measured on the day of administration and 1, 3, 7, and 14 days after administration (Fig. 1a). Thereafter, all animals were euthanized by exsanguination through the abdominal aorta under carbon dioxide anesthesia and were necropsied.

### Thirteen-week subchronic oral toxicity study

One day before treatment, a total of one hundred 6-week-old Sprague-Dawley rats were randomly assigned to four main groups (n = 20, 10 males and 10 females per group) and two recovery groups (n = 10, 5 males and 5 females per group). The four main groups were the control, low-dose, medium-dose, and high-dose groups (0, 800, 2000, and 5000 mg/kg/day, respectively), and the two recovery groups were the control and high-dose groups (0 and 5000 mg/kg/day, respectively). The recovery groups were included to investigate the reversibility of possible effects of HT047. HT047 was administered daily in a volume of 10 mL/kg body weight by oral gavage and the control groups received the vehicle (sterile water for injection; Choongwae Pharma Corp., Korea) in the same regimen. The duration of repeated dose toxicity study is recommended to be equal to or longer than the proposed clinical dosing period. We decided to conduct a 12-week clinical trial and thus a dosing period of 13 weeks was chosen. All rats received 13-week treatment and the rats in the recovery groups underwent 4 weeks of treatment-free observation period after the dosing period (Fig. 1b).

According to the ICH guideline, a dose of 2000 mg/kg/day is suggested for the maximum dose for repeated-dose toxicity study. However, a rat dose of 2000 mg/kg/day does not provide a 10-fold safety margin of the proposed human dose of HT047 (2250 mg/day). Since most stroke patients are elderly with multiple comorbid conditions and polypharmacy, it is important for a drug candidate to have a sufficient margin of safety. Therefore, a dose of 5000 mg/kg/day was chosen as the highest dose in this study.

#### Clinical signs

All animals were checked once daily for clinical signs and twice daily for moribund and mortality during the study period.

#### Body weight

The animals were weighed immediately before dosing on the first day of treatment (defined as Day 1), once a week thereafter, and on the day before necropsy. Body weight measured on the day of necropsy was excluded from the assessment because the rats were fasted.

#### Food consumption

Prior to the toxicity studies, 1-day food consumption between the time of group assignment and the start of treatment was measured. During the treatment and recovery periods, the mean daily food consumption was calculated as average of 7-day food consumption. At the 13^th^ week of treatment and 4^th^ week of recovery, the mean daily food consumption was calculated as average of 6-day food consumption.

#### Ophthalmologic examination

Ophthalmologic examination was performed on all animals in the main groups during the 13^th^ week of treatment and in the recovery groups during the 4^th^ week of recovery. Pupillary light reflex and anterior segment were observed macroscopically. After the application of a topical mydriatic agent (Isopto Atropine ophthalmic solution, Alcon, Lot No.: 13C07K), the anterior segment, transparent media, and ocular fundus were examined using an ophthalmoscope (All Pupil II, Keeler, UK).

#### Urinalysis

Urinalysis was performed on all animals in the main groups during the 13^th^ week of treatment and in the recovery groups during the 4^th^ week of recovery. Freshly voided urine (urine voided within 3 h) was collected, and the following indicators were measured using a Combur10Test®M stick (Roche, Germany) and a urine chemistry analyzer (cobas u 411, Roche, Germany): pH, protein, glucose, ketone body, bilirubin, and occult blood. Urine color and transparency were examined macroscopically, and urine sediment was examined microscopically. Concentrated urine (urine voided within approximately 24 h) was collected to measure volume and specific gravity using a measuring cylinder and a refractometer (Vet360, Reichert, USA), respectively.

#### Hematology

Prior to necropsy, all the animals were fasted for at least 18 h. On the day of necropsy, the animals were anesthetized with isoflurane, and blood samples were collected via the abdominal aorta. For hematology examination, approximately 1 mL of the blood sample was placed in a tube containing ethylenediaminetetraacetic acid and a hematology analyzer (ADVIA 2120i, Siemens, Germany) was used to measure the following: erythrocyte count, hemoglobin, hematocrit, mean corpuscular volume, mean corpuscular hemoglobin, mean corpuscular hemoglobin concentration, as well as platelet, leucocyte, neutrophil, lymphocyte, monocyte, eosinophil, basophil, and reticulocyte counts. For the coagulation test, approximately 2 mL of the blood sample was placed in a tube containing 3.2% sodium citrate and centrifuged at 3000 rpm for 10 min to obtain plasma. Prothrombin time and activated partial thromboplastin time were measured using a coagulation analyzer (Coapresta 2000, Sekisui, Japan).

#### Serum biochemistry

Blood samples were centrifuged at 3000 rpm for 10 min to obtain the serum. A biochemical analyzer (Hitachi 7180, Hitachi, Japan) was used to measure the following: alanine aminotransferase (ALT), aspartate aminotransferase (AST), alkaline phosphatase (ALP), blood urea nitrogen (BUN), creatinine, total bilirubin, total protein, albumin, albumin/globulin (A/G) ratio, total cholesterol, triglycerides, phosphorus, glucose, and calcium levels. An electrolyte analyzer (ILyte, Instrumentation Laboratory, USA) was used to measure chloride, sodium, and potassium levels.

#### Necropsy and organ weight

At the 92^nd^ and 120^th^ day for the main groups and recovery groups, respectively, all animals were euthanized by exsanguination through the abdominal aorta under isoflurane anesthesia and were necropsied. Detailed macroscopic examination was performed on the systemic organs and tissues. The wet weight of the following organs was measured and relative organ weight ratio with respect to fasting weight was calculated: the brain, pituitary, heart, lung, liver, spleen, kidney, adrenal, testis, prostate, ovary, and uterus. For organs in pairs, the weights of both organs were added together.

#### Histopathology

The following organs and tissues were extracted from all animals and fixed in 10% neutral buffered formalin: the brain, pituitary, thyroid, parathyroid, thymus, lung including bronchi, trachea, heart, liver, spleen, kidney, adrenal glands, salivary glands (submandibular, sublingual, and parotid glands), esophagus, stomach, duodenum, jejunum, ileum, cecum, colon, rectum, pancreas, testis, epididymis, prostate, seminal vesicle, ovary, uterus, vagina, urinary bladder, submandibular lymph node, mesenteric lymph node, eye (including optic nerve and harversian gland), inguinal mammary gland, inguinal skin, sternum (including bone marrow), femur (including bone marrow), tongue, and thoracic spinal cord. Testis and eyeballs were fixed in Davidson’s fixative. Histopathology examination was performed on: (1) all animals in the control and high-dose groups; (2) rats of the medium-dose and low-dose groups, if treatment-related changes were observed in the high-dose group; and (3) rats of the medium-dose and low-dose groups that showed changes in macroscopic necropsy findings. Tissue slides were prepared by the standard process, including dehydration, paraffin embedding, trimming, sectioning by microtome, and staining with hematoxylin and eosin. Bone tissues were decalcified using Calci-Clear™ Rapid solution (National diagnostics, USA). Residual organs and tissues were preserved in 10% neutral buffered formalin.

### Statistical analysis

Statistical analysis was performed using SAS (version 9.3, SAS Institute Inc., USA). For comparing two variances, the Folded F method was used to determine homoscedasticity. Student’s t-test and Aspin-Welch’s t-test were used for data with valid and nullified homoscedasticity, respectively. For comparing multiple variances, the Bartlett test was performed to determine homoscedasticity. Data with valid homoscedasticity was analyzed for significance with the one-way analysis of variance and further analyzed with the Dunnett’s multiple t-test. Data with nullified homoscedasticity was analyzed for significance with the Kruskal-Wallis test, and further analyzed with the Steel’s multiple test. Data are expressed as the mean ± standard deviation (SD). The significance level was set at *p* < 0.05.

## Results

### HPLC analysis

**Fig. 2:**
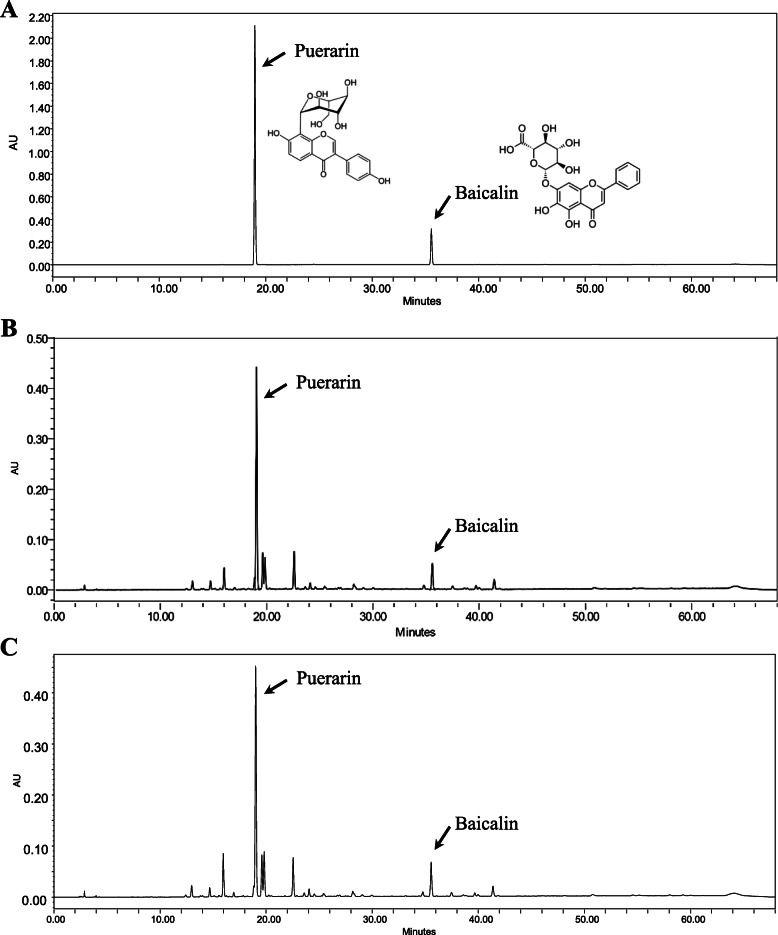
HPLC chromatograms of the standard mixture (**a**) and HT047 batches (**b–c**). Figure 1b and c shows the chromatograms of the batches used for the acute and subchronic toxicity studies, respectively. The absorbance was monitored at 254 nm

HT047 is standardized to contain no less than 76.10 and 19.80 mg/g puerarin and baicalin, respectively. For quality assurance, the contents of marker compounds in each of the batches used for this study were quantified using HPLC analysis. Figure 2 shows the HPLC chromatograms of a standard mixture (A) and HT047 used for this study (B and C). The puerarin and baicalin contents were 86.27 and 35.63 mg/g, respectively, in the batch used in the acute toxicity study (Fig. 2b), and 99.34 and 44.07 mg/g, respectively, in the batch used in the subchronic toxicity study (Fig. 2c). Both batches were confirmed to meet the established specifications. When HT047 was administered at a dose of 5000 mg/kg in the acute toxicity study, the dose equivalents of puerarin and baicalin were 431 and 178 mg/kg, respectively. In the subchronic toxicity study, the dose equivalents of puerarin in the 800, 2000, and 5000 mg/kg/day groups were 79, 199, and 497 mg/kg/day, respectively, and those of baicalin were 35, 88, and 220 mg/kg/day, respectively.

### Acute oral toxicity study

No death occurred in rats during the 14-day observation period. One day after dosing, test substance-colored stool was observed in all male and female rats treated with 5000 mg/kg HT047 and mucous stool was observed in 3 out of 5 males treated with 5000 mg/kg HT047. These findings were considered to be transient changes attributed to the test substance. No significant body weight change (Table 1) nor abnormal necropsy findings were observed in the rats.

**Table 1:** Body weight changes after a single dose of HT047

Sexes	Dose (mg/kg)	Days after dosing					Gain
		0^a^	1	3	7	14	0–14
Male	0^b^	153.1 ± 2.4	177.1 ± 4.2	197.4 ± 5.2	238.6 ± 7.1	307.8 ± 16.4	154.7 ± 14.7
	5000	154.8 ± 1.2	177.8 ± 1.8	198.7 ± 1.6	239.4 ± 2.4	310.0 ± 2.5	155.2 ± 2.2
Female	0^b^	132.9 ± 4.3	154.4 ± 5.6	165.3 ± 7.4	183.4 ± 9.1	213.6 ± 10.4	80.7 ± 10.8
	5000	133.5 ± 4.1	155.8 ± 3.4	166.6 ± 4.5	181.6 ± 6.0	208.5 ± 13.6	75.0 ± 9.8

^a^ The day of administration

^b^ Distilled water (10 mL/kg)

Data are expressed as mean ± SD in grams (n = 5 per group)

### Thirteen-week subchronic oral toxicity study

#### Clinical signs

No mortality was observed in any of the groups. Test substance-colored stool were observed in: 2–9 males on day 2–48, 8–10 males since day 49, 3–9 females on days 2–57, and all females since day 58 in the 800 mg/kg/day group; 7–10 males on days 2–28, all males since day 29, 7–9 females on days 2–20, and 9–10 females since day 21 in the 2000 mg/kg/day group; and 14 males on day 2, all males since day 3, and all females since day 2 in the 5000 mg/kg/day group. During the recovery period, test substance-colored stool was observed up to the 2^nd^ day of recovery in male and female rats of the 5000 mg/kg/day group. These findings were not considered as toxic changes. Salivation was observed sporadically in males and females in the 5000 mg/kg/day group; however, it was not considered toxicologically relevant because it was a transient phenomenon. Chromaturia was observed on the 87^th^ day in 1 and 3 males in the 800 and 5000 mg/kg/day groups, respectively. On the contrary, 1–5 females in the 5000 mg/kg/day group showed sporadic chromaturia starting from day 23. These observations were due to urine being colored by the test substance and were not considered toxicologically relevant. A mass was observed in the left shoulder and left lumbar region of a female in the 2000 mg/kg/day group, starting from the 49^th^ days. Histopathological examination confirmed the mass to be a mammary gland adenocarcinoma, which was considered to have occurred spontaneously. Other clinical signs observed included crust formation in the right hindlimb due to toenail damage (1 male in the 2000 mg/kg/day group), soiled perineal region (1 male and 2 females in the 5000 mg/kg/day group), and loss of fur (3 females in the 5000 mg/kg/day group). These clinical signs were not considered treatment-related, because they appeared transiently or only in 1 or 3 animals.

#### Body weight

Males in the 800 mg/kg/day group showed weight loss tendency or significant weight loss at 3–13 weeks compared to the control group (Table 2). Males in the 2000 and 5000 mg/kg/day groups showed a weight loss tendency at 10–13 weeks (not significant). Whereas females in the 800, 2000, and 5000 mg/kg/day group showed significant weight loss at 4–13, 2–13, and 2–13 weeks, respectively. Owing to the weight difference during the administration period, females in the 5000 mg/kg/day recovery group showed significant weight loss on the first day and the 1^st^ week of the recovery period, compared to that observed in the control group, and showed subsequent recovery (Table 3).

**Table 2:** Body weight changes during the 13-week administration of HT047

Weeks	Male dose (mg/kg/day)				Female dose (mg/kg/day)			
	0^a^	800	2000	5000	0^a^	800	2000	5000
0^b^	188.1 ± 5.2^c^	188.1 ± 6.2	189.4 ± 10.1	188.9 ± 6.1	172.5 ± 6.6	171.8 ± 8.6	172.7 ± 10.5	169.9 ± 8.1
1	252.9 ± 10.9	251.1 ± 10.8	254.0 ± 13.8	252.2 ± 9.9	196.8 ± 10	190.3 ± 10.1	190.6 ± 10.5	186.6 ± 9.8
2	309.5 ± 17.9	304.3 ± 22.0	311.0 ± 18.7	310.1 ± 11.5	219.3 ± 13.9	209.9 ± 9.6	204.1 ± 10.0^*^	203.6 ± 15.3^**^
3	351.6 ± 25.4	341.2 ± 30.0	354.1 ± 26.9	354.0 ± 17.7	239.9 ± 17.4	225.1 ± 14.4	215.3 ± 16.1^**^	217.2 ± 18.2^**^
4	388.2 ± 30.8	370.0 ± 34.2	387.6 ± 30.2	389.5 ± 22.9	261.8 ± 18.6	235 ± 10.1^**^	226.6 ± 15.6^**^	226.3 ± 15.9^**^
5	421.3 ± 34.6	394.7 ± 32.4	420.4 ± 32.8	424.1 ± 26.3	277.5 ± 20.3	246.1 ± 12.5^**^	236.3 ± 15.9^**^	238.9 ± 19.4^**^
6	448.8 ± 37.8	419.4 ± 36.5	448.7 ± 32.2	449.3 ± 31.9	292.6 ± 23.4	254.4 ± 14.7^**^	248.5 ± 13.6^**^	246.9 ± 21^**^
7	470.0 ± 44.6	438.5 ± 37.0	474.3 ± 30.8	473.2 ± 35.2	302.2 ± 26.1	262.5 ± 14.6^**^	258.9 ± 15.0^**^	255.5 ± 19.8^**^
8	492.6 ± 47.1	454.2 ± 39.3	491.2 ± 32.4	490.8 ± 38.7	310.4 ± 25.9	264.9 ± 12.9^**^	263.6 ± 17.4^**^	259.4 ± 21.8^**^
9	511.5 ± 49.7	465.4 ± 38.3*	510.6 ± 34.0	505.3 ± 42.9	316.4 ± 28.2	271.1 ± 15.8^**^	266.2 ± 19.5^**^	260.9 ± 22.7^**^
10	526.1 ± 52.8	473.7 ± 38.1*	520.0 ± 37.7	513.4 ± 45.9	319.9 ± 29.6	274.6 ± 15.5^**^	272.6 ± 20.5^**^	264.3 ± 22.8^**^
11	538.4 ± 57.7	484.5 ± 37.5	527.2 ± 43.0	523.0 ± 47.1	326.4 ± 30.7	275.2 ± 13.7^**^	277.2 ± 22.5^**^	266.4 ± 21^**^
12	551.6 ± 59.4	492.3 ± 37.2^*^	534.8 ± 44.2	531.8 ± 50.0	328.1 ± 32.1	278.8 ± 15.7^**^	281.6 ± 21.5^**^	269.6 ± 22.8^**^
13	553.5 ± 59.4	496.5 ± 38.2	540.8 ± 46.7	535.8 ± 50.9	329.7 ± 30.6	281.5 ± 14.5^**^	282.0 ± 24.5^**^	272.0 ± 22.7^**^

^a^ Distilled water (10 mL/kg/day)

^b^ The day of first treatment was designated week 0

^c^ Data are expressed as mean ± SD in grams (n = 15 for the control and 5000 mg/kg/day groups; n = 10 for the 800 and 2000 mg/kg/day groups)

* *p* < 0.05 and ** *p* < 0.01 vs. control by one-way ANOVA with post-hoc Dunnett’s t-test

**Table 3:** Body weight changes during the recovery period in the subchronic toxicity study of HT047

Weeks	Male dose (mg/kg/day)		Female dose (mg/kg/day)	
	0^a^	5000	0^a^	5000
13^b^	540.4 ± 59.8^c^	530.8 ± 63.6	323.3 ± 28.4	263.8 ± 33.0^#^
14	547.8 ± 61.0	533.4 ± 66.5	330.7 ± 31.3	281.8 ± 34.6^#^
15	558.4 ± 61.5	547.4 ± 70.3	334.9 ± 35.2	293.1 ± 36.5
16	569.3 ± 61.6	564.0 ± 72.0	339.8 ± 34.4	300.1 ± 40.0
17	576.8 ± 64.4	567.1 ± 72.9	341.8 ± 33.1	304.9 ± 41.3

^a^ Distilled water (10 mL/kg/day)

^b^ The first day of the recovery period was designated week 13

^c^ Data are expressed as mean ± SD in grams (n = 5 per group)

# *p* < 0.05 vs. control by Student’s *t*-test

#### Food consumption

Males in the 800 mg/kg/day group at the 9^th^ week, females in the 800 mg/kg/day group at 1–8 weeks and the 11^th^ week, females in the 2000 mg/kg/day group at 1–6 weeks and the 8^th^ week, and females in the 5000 mg/kg/day group at 1–9 weeks showed significantly lower food consumption than the controls (Table 4). However, the decreases were slight and had no toxicological significance. During the recovery period, female rats in the 5000 mg/kg/day group showed a significantly higher food consumption at 14–15 weeks than those of the control group (Table 5).

**Table 4:** Food consumption during the 13-week administration of HT047

Weeks	Male dose (mg/kg/day)				Female dose (mg/kg/day)			
	0^a^	800	2000	5000	0^a^	800	2000	5000
0^b^	24.7 ± 2.2^c^	24.8 ± 1.5	25.5 ± 2.9	25.4 ± 1.9	22.0 ± 2.1	21.3 ± 2.7	21.2 ± 2.0	20.3 ± 2.3
1	28.2 ± 2.5	27.7 ± 1.8	28.1 ± 2.3	27.6 ± 2.2	22.3 ± 1.6	20.1 ± 1.4^**^	19.9 ± 1.5^**^	18.8 ± 3.0^**^
2	31.7 ± 3.2	30.7 ± 3.0	31.8 ± 3.1	31.9 ± 2.3	24.2 ± 2.2	21.8 ± 1.6^*^	19.9 ± 1.7^**^	19.8 ± 2.8^**^
3	32.2 ± 3.8	31.2 ± 3.8	32.4 ± 2.7	33.4 ± 3.3	25.3 ± 2.7	22.4 ± 3.0^*^	20.7 ± 2.3^**^	20.4 ± 2.9^**^
4	32.5 ± 3.7	30.9 ± 3.3	32.5 ± 2.7	34.5 ± 3.0	26.2 ± 2.7	21.5 ± 1.9^**^	20.5 ± 1.9^**^	20.8 ± 3.5^**^
5	33.4 ± 3.8	31.3 ± 2.7	34.2 ± 3.3	35.5 ± 3.2	26.8 ± 3.1	22.9 ± 1.9^##^	21.9 ± 1.2^##^	22.3 ± 3.8^##^
6	33.5 ± 4.1	31.4 ± 2.6	35.3 ± 3.2	35.3 ± 3.5	26.5 ± 3.1	22.4 ± 2.2^##^	23.1 ± 1.6^##^	22.4 ± 4.0^#^
7	33.7 ± 4.4	31.3 ± 2.5	35.7 ± 2.7	35.8 ± 4.0	25.6 ± 3.4	22.1 ± 1.3^##^	23.0 ± 2.3	22.2 ± 3.2^#^
8	33.5 ± 4.4	30.7 ± 2.8	34.7 ± 2.9	34.9 ± 3.8	24.9 ± 2.8	20.9 ± 2.0^**^	22.2 ± 2.0^*^	21.7 ± 3.2^**^
9	33.7 ± 4.4	29.8 ± 2.5^*^	33.3 ± 3.6	35.0 ± 3.8	24.2 ± 3.1	21.8 ± 2.2	22.3 ± 1.8	21.1 ± 3.2^*^
10	33.2 ± 4.7	29.8 ± 2.9	32.9 ± 3.0	34.2 ± 4.1	23.9 ± 2.5	21.6 ± 1.5	22.4 ± 3.0	21.6 ± 3.0
11	33.2 ± 5.0	30.1 ± 2.5	32.4 ± 3.5	33.8 ± 3.7	23.3 ± 2.4	20.1 ± 1.4^##^	21.7 ± 2.5	21.3 ± 3.7
12	32.7 ± 5.2	30.1 ± 2.5	31.4 ± 3.7	32.6 ± 4.0	22.3 ± 2.4	19.6 ± 2.0	21.3 ± 3.1	20.8 ± 3.1
13	31.2 ± 4.3	28.8 ± 1.9	31.5 ± 3.1	32.0 ± 4.4	21.1 ± 2.1	19.7 ± 2.3	20.7 ± 2.8	20.3 ± 4.1

^a^ Distilled water (10 mL/kg/day)

^b^ The day of first treatment was designated week 0

^c^ Values are expressed as grams per rat per day (g/rat/day) (n = 15 for the control and 5000 mg/kg/day groups; n = 10 for the 800 and 2000 mg/kg/day groups)

* *p* < 0.05 and ** *p* < 0.01 vs. control by one-way ANOVA with post-hoc Dunnett’s t-test

# *p* < 0.05 and ## *p* < 0.01 vs. control by Kruskal-Wallis test with post-hoc Steel's test

**Table 5:** Food consumption during the recovery period in the subchronic toxicity study of HT047

Weeks	Male dose (mg/kg/day)		Female dose (mg/kg/day)	
	0^a^	5000	0^a^	5000
14	31.2 ± 3.7^b^	35.3 ± 6.7	23.0 ± 2.2	29.4 ± 2.6^**^
15	31.3 ± 4.0	34.6 ± 6.6	21.7 ± 2.7	27.0 ± 4.0^*^
16	32.3 ± 3.9	34.7 ± 4.8	22.4 ± 2.6	26.0 ± 4.5
17	30.0 ± 3.7	32.6 ± 5.7	20.3 ± 2.4	23.2 ± 3.9

^a^ Distilled water (10 mL/kg/day)

^b^ Values are expressed as grams per rat per day (g/rat/day) (n = 5 per group)

* *p* < 0.05 and ** *p* < 0.01 vs. control by Student’s *t*-test

#### Ophthalmologic examination

No ophthalmological abnormality was observed in any of the rats in the main and recovery groups (data not shown).

#### Urinalysis

Urine volume was significantly increased in males treated with 2000 mg/kg/day HT047 compared to the control group (15.5 ± 5.1 vs. 10.6 ± 2.6 mL, *p* < 0.05), while no significant change was observed in males treated with 800 mg/kg/day (11.5 ± 4.1 mL) and 5000 mg/kg/day (10.9 ± 3.8 mL). There were no differences in urine volume between the female groups (data not shown). In addition, the changes in urine color, transparency, pH, protein, ketone body, bilirubin, occult blood, and specific gravity level observed in the main groups were slight and non-significant (Table 6). In urine sediment examination, no cast, epithelial cell, leukocyte, or erythrocyte was present in the urine of any rats. The recovery groups showed no treatment-related effect (data not shown).

**Table 6:** Urinalysis findings after 13-week administration of HT047

Parameters	Results	Male dose (mg/kg/day)				Female dose (mg/kg/day)			
		0^a^	800	2000	5000	0^a^	800	2000	5000
Color	Pale yellow	5^b^	1	0	0	3	3	0	0
	Yellow	5	9	10	10	7	7	9	7
	Amber	0	0	0	0	0	0	1	3
Transparency	Clear	10	10	10	10	10	8	5	4
	Mild turbidity	0	0	0	0	0	1	4	1
	Turbidity	0	0	0	0	0	1	1	5
pH	7	3	0	3	0	3	0	2	0
	8	7	10	6	9	6	7	5	5
	9	0	0	1	1	1	3	3	5
Protein	Negative	9	9	5	6	8	8	8	4
	25 mg/dL	1	1	4	4	2	1	2	6
	75 mg/dL	0	0	1	0	0	0	0	0
	150 mg/dL	0	0	0	0	0	1	0	0
Glucose	Normal	10	10	10	10	10	10	10	10
Ketone body	Negative	6	6	2	0	6	7	1	0
	5 mg/dL	4	3	6	3	4	1	6	5
	15 mg/dL	0	1	2	5	0	2	2	5
	50 mg/dL	0	0	0	2	0	0	1	0
Bilirubin	Negative	10	10	10	10	9	9	9	10
	1 mg/dL	0	0	0	0	1	1	1	0
Occult blood	Negative	9	10	9	10	10	10	10	9
	10 Ery/μL	1	0	0	0	0	0	0	0
	50 Ery/μL	0	0	1	0	0	0	0	1
Specific gravity	1.000 ~ 1.010	0	0	0	0	0	1	0	0
	1.021 ~ 1.030	0	0	1	1	1	2	1	1
	1.031 ~ 1.040	1	3	4	0	0	2	4	3
	1.041 ~ 1.050	6	2	3	2	6	2	3	0
	1.051 ~ 1.060	3	5	1	3	2	1	0	5
	> 1.060	0	0	1	4	1	2	2	1

^a^ Distilled water (10 mL/kg/day)

^b^ The number of rats in each group (n = 10 per group)

#### Hematology

Statistically significant changes in male rats included the following (Table 7): a decrease in reticulocyte (800, 2000, and 5000 mg/kg/day) and eosinophils (5000 mg/kg/day) levels, as well as an increase in prothrombin time (5000 mg/kg/day). In female rats, the significant changes were as follows: a decrease in erythrocyte, hemoglobin, hematocrit, and eosinophil levels (5000 mg/kg/day group), a decrease in neutrophil and monocyte levels (800 and 5000 mg/kg/day groups), an increase in lymphocyte levels (800 and 5000 mg/kg/day groups), and an increase in prothrombin time (800, 2000, and 5000 mg/kg/day groups). In the recovery group, there were no significant changes, except for a decrease in basophil levels in males of the 5000 mg/kg/day group (0.3 ± 0.0 vs. 0.2 ± 0.1%, *p* < 0.05).

**Table 7:** Summary of selected^a^ hematological findings after 13-week administration of HT047

Parameters	Units	Male dose (mg/kg/day)				Female dose (mg/kg/day)			
		0^**b**^	800	2000	5000	0^**b**^	800	2000	5000
RBC	× 10^6^ cells/μL	8.72 ± 0.25	8.80 ± 0.41	8.81 ± 0.36	8.58 ± 0.35	7.95 ± 0.37	7.85 ± 0.32	7.93 ± 0.42	7.42 ± 0.53^*^
Hemoglobin	g/dL	15.1 ± 0.3	15.4 ± 0.7	15.4 ± 0.3	15.3 ± 0.5	14.6 ± 0.7	14.7 ± 0.3	14.6 ± 0.9	13.7 ± 0.6^#^
Hematocrit	%	45.3 ± 1.2	46.2 ± 1.9	45.6 ± 1.8	45.4 ± 1.6	42.4 ± 2.2	42.1 ± 1.2	41.8 ± 2.6	39.5 ± 2.0^**^
Reticulocytes	%	2.73 ± 0.46	2.19 ± 0.54^*^	2.21 ± 0.31^*^	2.13 ± 0.36^**^	2.05 ± 0.35	2.17 ± 0.3	1.86 ± 0.29	2.29 ± 0.63
Neutrophils	%	20.7 ± 6.6	19.1 ± 6.8	17.4 ± 4.4	16.3 ± 6.2	19.2 ± 6.0	13.0 ± 4.2^*^	18.4 ± 5.4	13.7 ± 3.1^*^
Lymphocytes	%	74.7 ± 6.7	76.6 ± 6.5	78.5 ± 4.7	79.6 ± 6.5	75.6 ± 6.1	83.5 ± 4.4^**^	76.9 ± 5.5	82.6 ± 3.5^**^
Monocytes	%	2.3 ± 0.5	2.0 ± 1.0	1.8 ± 0.6	1.8 ± 0.5	2.4 ± 0.8	1.5 ± 0.5^*^	1.9 ± 0.9	1.6 ± 0.5^*^
Eosinophils	%	1.2 ± 0.2	1.0 ± 0.4	1.3 ± 0.2	0.8 ± 0.2^##^	1.7 ± 0.5	1.1 ± 0.3	1.6 ± 0.8	1.0 ± 0.3^#^
Prothrombin time	sec	17.8 ± 0.4	18.0 ± 0.4	18.0 ± 1.0	19.5 ± 1.1^##^	17.3 ± 0.7	18.7 ± 1.0^**^	18.6 ± 1.2^**^	19.0 ± 0.7^**^

^a^ Only parameters with statistical significance are shown in table. RBC, red blood cell

^b^ Distilled water (10 mL/kg/day)

* *p* < 0.05 and ** *p* < 0.01 vs. control by one-way ANOVA with post-hoc Dunnett’s t-test

# *p* < 0.05 and ## *p* < 0.01 vs. control by Kruskal-Wallis test with post-hoc Steel's test

Data are presented as mean ± SD (n = 10 per group)

#### Serum biochemistry

The significant changes were as follows (Table 8): (1) an increase in alkaline phosphatase levels (females of the 800, 2000, and 5000 mg/kg/day groups); (2) a decrease in total cholesterol levels (both sexes of the 800, 2000, and 5000 mg/kg/day groups); (3) a decrease in total protein levels (males of the 800 mg/kg/day group); (4) a decrease in the A/G ratio (females of the 800, 2000, and 5000 mg/kg/day groups); (5) a decrease in calcium levels (males of the 800 mg/kg/day groups); and (6) a decrease in chloride levels (males of the 5000 mg/kg/day group and females of the 2000 and 5000 mg/kg/day groups).

**Table 8:** Serum biochemical findings after 13-week administration of HT047

Parameters	units	Male dose (mg/kg/day)				Female dose (mg/kg/day)			
		0^a^	800	2000	5000	0^a^	800	2000	5000
ALT	U/L	30.5 ± 5.7	31.7 ± 6.9	29.9 ± 4.6	29.2 ± 10.8	24.0 ± 6.3	25.4 ± 8.0	23.1 ± 7.9	22.2 ± 6.4
AST	U/L	77.0 ± 10.3	80.4 ± 10.7	76.7 ± 12.8	67.6 ± 12.2	68.9 ± 11.3	74.8 ± 18.0	83.9 ± 23.2	78.7 ± 20.8
ALP	U/L	279.5 ± 54.7	312.5 ± 54.2	315 ± 89.6	344.5 ± 70.7	131.3 ± 25.2	180.7 ± 37.2^*^	214.6 ± 51.0^**^	200.7 ± 54.2^**^
Glucose	mg/dL	126 ± 14	127 ± 15	124 ± 15	124 ± 18	148 ± 7	146 ± 18	150 ± 14	132 ± 11^*^
BUN	mg/dL	12.1 ± 1.4	11.5 ± 1.6	11.3 ± 1.1	10.9 ± 1.4	13.0 ± 1.9	12.9 ± 1.7	13.8 ± 2.6	14.6 ± 3.2
Creatinine	mg/dL	0.43 ± 0.05	0.43 ± 0.04	0.45 ± 0.03	0.44 ± 0.05	0.47 ± 0.05	0.44 ± 0.03	0.47 ± 0.06	0.47 ± 0.03
Total bilirubin	mg/dL	0.06 ± 0.01	0.06 ± 0.01	0.06 ± 0.03	0.06 ± 0.01	0.11 ± 0.03	0.13 ± 0.04	0.12 ± 0.03	0.13 ± 0.02
TC	mg/dL	84 ± 13	53 ± 17^##^	47 ± 24^#^	28 ± 5^##^	87 ± 21	48 ± 24^**^	46 ± 17^**^	45 ± 23^**^
TG	mg/dL	61 ± 19	46 ± 21	84 ± 45	87 ± 37	23 ± 7	22 ± 11	25 ± 13	29 ± 11
Total protein	g/dL	6.1 ± 0.2	5.8 ± 0.2*	6.0 ± 0.2	6.0 ± 0.2	6.2 ± 0.3	6.5 ± 0.5	6.4 ± 0.4	6.5 ± 0.6
Albumin	g/dL	2.4 ± 0.1	2.4 ± 0.1	2.4 ± 0.1	2.4 ± 0.1	2.8 ± 0.2	2.8 ± 0.3	2.7 ± 0.2	2.7 ± 0.3
A/G ratio	–	0.65 ± 0.04	0.69 ± 0.05	0.66 ± 0.05	0.65 ± 0.04	0.83 ± 0.06	0.76 ± 0.05^*^	0.74 ± 0.04^**^	0.71 ± 0.05^**^
P	mg/dL	6.14 ± 0.59	6.01 ± 0.66	6.30 ± 0.52	6.73 ± 0.56	4.24 ± 0.41	4.97 ± 0.48	4.65 ± 0.94	4.89 ± 0.69
Ca	mg/dL	10.1 ± 0.3	9.7 ± 0.1^**^	10.1 ± 0.2	10.0 ± 0.3	9.9 ± 0.2	9.9 ± 0.3	10.4 ± 1.8	9.9 ± 0.4
Na	mmol/L	141.4 ± 1.1	141.4 ± 1.2	140.9 ± 0.9	140.3 ± 0.9	140.8 ± 1.6	141.7 ± 0.6	140.0 ± 1.2	140.2 ± 0.8
K	mmol/L	4.27 ± 0.16	4.25 ± 0.27	4.29 ± 0.23	4.20 ± 0.20	4.05 ± 0.31	4.26 ± 0.16	4.14 ± 0.46	4.22 ± 0.35
Cl	mmol/L	108.0 ± 1.2	107.7 ± 1.5	106.9 ± 1.1	106.5 ± 0.9^*^	108.9 ± 1.3	108.4 ± 1.3	106.2 ± 1.3^**^	106.6 ± 1.9^**^

^a^ Distilled water (10 mL/kg/day)

*A/G* Albumin/globulin, *ALP* Alkaline phosphatase, *ALT* Alanine aminotransferase, *AST* Aspartate aminotransferase, *BUN* Blood urea nitrogen, *Ca* Calcium, *Cl* Chloride, *K* Potassium, *Na* Sodium, *P* Phosphorus, *TC* Total cholesterol, *TG* Triglycerides

* *p* < 0.05 and ** *p* < 0.01 vs. control by one-way ANOVA with post-hoc Dunnett’s t-test

# *p* < 0.05 and ## *p* < 0.01 vs. control by Kruskal-Wallis test with post-hoc Steel's test

Data are presented as mean ± SD (n = 10 per group)

Males of the recovery groups showed no significant changes in serum biochemical indicators (data not shown). Females in the recovery groups showed no significant changes except in the following parameters: (1) an increase in total cholesterol levels (83 ± 12 vs. 114 ± 9 mg/dL, *p* < 0.01); (2) an increase in total protein levels (6.3 ± 0.3 vs. 6.8 ± 0.3, *p* < 0.05); and (3) an increase in calcium levels (10.1 ± 0.3 vs. 10.6 ± 0.2 mg/dL, *p* < 0.05).

#### Absolute and relative organ weight

Significant changes in organ weight included the following (Table 9): (1) an increase in the relative brain weight (males treated at 800 mg/kg/day and females treated at 800, 2000, and 5000 mg/kg/day); (2) an increase in the absolute pituitary weight (females treated at 2000 and 5000 mg/kg/day); (3) a decrease in the absolute heart weight (males and females treated at 800 mg/kg/day); (4) an increase in the relative heart weight (females treated at 2000 and 5000 mg/kg/day); (5) a decrease in the absolute lung weight (females treated at 800 and 5000 mg/kg/day); (6) an increase in the relative lung weight (males treated at 800 mg/kg/day and females treated at 800, 2000, and 5000 mg/kg/day); (7) an increase in the relative liver weight (males treated at 5000 mg/kg/day and females treated at 800, 2000, and 5000 mg/kg/day); (8) an increase in the relative spleen weight (females treated at 2000 and 5000 mg/kg/day); (9) an increase in the absolute kidney weight (males treated at 5000 mg/kg/day); (10) an increase in the relative kidney weight (both sex in the 5000 mg/kg/day groups); and (11) an increase in the relative adrenal weight (females in the 800, 2000, and 5000 mg/kg/day groups). Males of the recovery groups showed no significant changes in organ weight (data not shown). Females of the recovery groups showed no significant changes, except for increases in the relative weights of the pituitary (4.6 ± 1.0 vs. 6.1 ± 1.1 mg/100 g body weight, *p* < 0.05), liver (2.35 ± 0.14 vs. 2.97 ± 0.10 g/100 g body weight, *p* < 0.01), and kidney (0.60 ± 0.04 vs. 0.71 ± 0.09 g/100 g body weight, *p* < 0.05).

**Table 9:** Summary of selected^a^ absolute and relative organ weights in the subchronic toxicity study of HT047

Organs	Units	Male dose (mg/kg/day)				Female dose (mg/kg/day)			
		0^**b**^	800	2000	5000	0^**b**^	800	2000	5000
Body weight^c^	g	532.6 ± 59.9	470.0 ± 36.2^*^	512.1 ± 44	508.5 ± 42.8	314.6 ± 31.3	262.2 ± 13.6^**^	263.0 ± 23.4^**^	252.9 ± 18.9^**^
Brain	g	2.10 ± 0.07	2.10 ± 0.07	2.14 ± 0.11	2.12 ± 0.06	1.94 ± 0.08	1.93 ± 0.09	1.95 ± 0.08	1.93 ± 0.07
	g/100 g BW	0.40 ± 0.04	0.45 ± 0.03^**^	0.42 ± 0.03	0.42 ± 0.03	0.62 ± 0.08	0.74 ± 0.04^**^	0.75 ± 0.08^**^	0.77 ± 0.07^**^
Pituitary	mg	12.8 ± 1.9	11.1 ± 0.8	12.1 ± 1.5	13.6 ± 2.0	16.9 ± 2.8	14.9 ± 1.7	13.7 ± 1.7^**^	13.8 ± 1.8^**^
	mg/100 g BW	2.4 ± 0.2	2.4 ± 0.3	2.4 ± 0.3	2.7 ± 0.4	5.4 ± 1.0	5.7 ± 0.6	5.2 ± 0.7	5.4 ± 0.6
Heart	g	1.49 ± 0.16	1.28 ± 0.10^**^	1.39 ± 0.18	1.43 ± 0.10	0.99 ± 0.09	0.87 ± 0.10^*^	0.92 ± 0.06	0.93 ± 0.10
	g/100 g BW	0.28 ± 0.02	0.27 ± 0.01	0.27 ± 0.03	0.28 ± 0.02	0.32 ± 0.02	0.33 ± 0.03	0.35 ± 0.03^*^	0.37 ± 0.03^**^
Lung	g	1.54 ± 0.14	1.53 ± 0.10	1.60 ± 0.13	1.54 ± 0.09	1.25 ± 0.07	1.16 ± 0.07^*^	1.18 ± 0.10	1.16 ± 0.09^*^
	g/100 g BW	0.29 ± 0.02	0.32 ± 0.02^*^	0.31 ± 0.03	0.30 ± 0.03	0.40 ± 0.04	0.45 ± 0.03^*^	0.45 ± 0.04^**^	0.46 ± 0.02^**^
Liver	g	13.87 ± 2.06	11.99 ± 1.28	14.36 ± 2.05	15.81 ± 1.90	7.67 ± 0.67	7.37 ± 0.83	7.89 ± 0.64	8.39 ± 0.74
	g/100 g BW	2.60 ± 0.15	2.55 ± 0.18	2.80 ± 0.22	3.10 ± 0.19^**^	2.45 ± 0.23	2.81 ± 0.26^*^	3.01 ± 0.29^**^	3.32 ± 0.28^**^
Spleen	g	0.92 ± 0.10	0.79 ± 0.12	0.85 ± 0.12	0.86 ± 0.09	0.55 ± 0.08	0.51 ± 0.06	0.54 ± 0.05	0.55 ± 0.09
	g/100 g BW	0.17 ± 0.02	0.17 ± 0.02	0.17 ± 0.02	0.17 ± 0.03	0.18 ± 0.03	0.20 ± 0.01	0.21 ± 0.02^*^	0.22 ± 0.03^**^
Kidney	g	3.24 ± 0.45	2.98 ± 0.36	3.25 ± 0.33	3.69 ± 0.41^*^	1.91 ± 0.17	1.77 ± 0.13	1.92 ± 0.22	1.79 ± 0.12
	g/100 g BW	0.61 ± 0.05	0.63 ± 0.06	0.64 ± 0.04	0.73 ± 0.06^**^	0.62 ± 0.08	0.68 ± 0.05	0.74 ± 0.13	0.71 ± 0.04^#^
Adrenal	mg	61.9 ± 8.0	53.7 ± 7.3	57.1 ± 6.8	69.1 ± 17.1	65.1 ± 9.0	72.1 ± 16.9	81.9 ± 11.9	72.3 ± 19.4
	mg/100 g BW	11.8 ± 2.2	11.5 ± 1.8	11.3 ± 1.8	13.5 ± 2.8	20.9 ± 3.7	27.4 ± 5.1^*^	31.5 ± 6.2^**^	28.5 ± 7.0^*^

^a^ Only parameters with statistical significance are shown in table

^b^ Distilled water (10 mL/kg/day)

^c^ Fasting weight on the 92^nd^ day before sacrifice. BW, body weight

* *p* < 0.05 and ** *p* < 0.01 vs. control by one-way ANOVA with post-hoc Dunnett’s t-test

# *p* < 0.05 vs. control by Kruskal-Wallis test with post-hoc Steel's test

Data are presented as mean ± SD (n = 10 per group)

#### Necropsy

No abnormal necropsy finding was observed in males of the main groups. Loss of hair from the skin was observed in 2/10 females of the 5000 mg/kg/day group. A black focal lesion in the glandular stomach was observed in 1/10 females of the 800 mg/kg/day group. Mass in the subcutaneous tissue of the back and left inguinal area were observed in 1/10 females of the 2000 mg/kg/day group. In the recovery groups, 2/5 males of the control group showed abnormal findings, including diverticulum of jejunum (1/5) and two red focal lesions in the glandular stomach (1/5). Hair loss was observed in 1/5 females of the 5000 mg/kg/day recovery group. These findings were not considered treatment-related because they occurred spontaneously or sporadically.

#### Histopathology

**Fig. 3:**
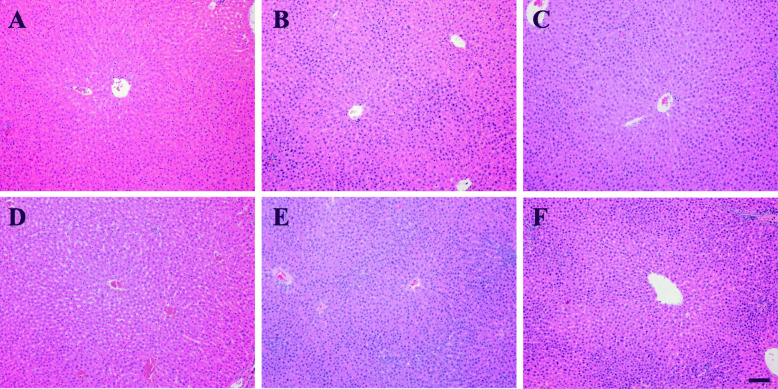
Liver histology of rats in the control and HT047 groups. Representative H&E stained photomicrographs of male (**a–c**, top row) and female (**d–f**, bottom row) rats. **a** Control male group showing normal hepatocytes. **b–c** Males of the 5000 mg/kg/day group showing minimal (**b**) or mild (**c**) centrilobular hepatocellular hypertrophy. **d** Control female group showing normal hepatocytes. **e–f** Centrilobular hepatocellular hypertrophy was observed in females of the 2000 mg/kg/day group (**e**, graded as minimal) and 5000 mg/kg/day group (**f**, graded as mild). Scale bar = 100 μm

Centrilobular hepatocellular hypertrophy was observed in 3/10 males of the 5000 mg/kg/day group and the severity was graded minimal in one male and mild in two males (Fig. 3a–c). Centrilobular hepatocellular hypertrophy was also observed in 2/10 females of the 2000 mg/kg/day group (graded minimal) and in 7/10 females of the 5000 mg/kg/day group (graded minimal in 2 females and mild in 5 females)(Fig. 3d–f). Rats of the recovery groups showed no centrilobular hepatocellular hypertrophy. The subcutaneous mass observed in the left inguinal area and lumbar spine of one female of the 2000 mg/kg/day group was confirmed to be mammary gland adenocarcinoma. This was considered a sporadic change unrelated to the treatment, because it occurred only in a single case in the medium-dose group. All other findings were not considered toxicologically relevant because they occurred spontaneously or sporadically and were naturally occurring changes commonly found in Sprague-Dawley rats at that age.

## Discussion

The present study was conducted to evaluate the potential toxicity of HT047 after single and 13-week repeated oral administration in rats. The results of the acute and subchronic toxicity studies did not reveal any toxicologically significant changes in rats treated with HT047 at any doses.

In the acute oral toxicity study, HT047 at a dose of 5000 mg/kg did not cause any mortality or treatment-related effects on clinical signs, body weight, and necropsy. Under the experimental conditions of this study, the oral ALD of HT047 was found to be greater than 5000 mg/kg in male and female rats. According to U.S. EPA guidelines, HT047 is classified as practically nontoxic.

In the subchronic oral toxicity study, HT047 at doses of 800, 2000, and 5000 mg/kg/day caused no death and no treatment-related adverse effects on food consumption; organ weight; ophthalmologic, urinalysis, and hematological parameters; and necropsy findings in rats of both sexes. There were some treatment-related changes in clinical signs (test substance-colored stool and urine), body weight (weight reduction), and serum biochemistry (decreased cholesterol level) and histopathological (centrilobular hepatocellular hypertrophy) parameters; however, these changes resolved during the recovery period.

HT047 reduced the body weight gain by 16% in males at a dose of 800 mg/kg/day (not significant) and by 30, 31, and 35% in females at dose of 800, 2000, and 5000 mg/kg/day, respectively (all *p* < 0.01). These changes were not dose-dependent and considered to be due to the reduction in fat accumulation by *P. lobata* and *S. baicalensis* [26, 27], attributed to their major active components puerarin and baicalin [26, 28–30]; therefore, such weight loss is not considered toxicologically significant. Serum biochemistry revealed that HT047 significantly reduced the total cholesterol level by 36, 44, and 67% in males at doses of 800, 2000, and 5000 mg/kg/day, respectively, and by 45, 48, and 48% in females at doses of 800, 2000, and 5000 mg/kg/day, respectively. The change in total cholesterol level was considered to be due to the cholesterol-lowering effects of *P. lobata* and *S. baicalensis* [26, 27], which are attributed to their major active components, puerarin and baicalin [28–31]. Thus, it was determined that the change in total cholesterol level was of no toxicological significance. The other significant serum biochemical findings were within normal ranges [32] with no distinct dose-dependence, and unaccompanied by correlative findings; thus, these findings were also considered toxicologically insignificant. The severity of centrilobular hepatocellular hypertrophy observed in histopathology ranged from minimal to mild in affected rats and this change was determined to be an adaptive response to increased hepatic metabolic activities associated with the treatment [33, 34]. Hence, all of these treatment-related changes were concluded to be not toxicologically significant.

The statistically significant changes in urinalysis, hematology, and organ weight parameters remained within normal ranges [32], were not dose-dependent, and were unaccompanied by correlative findings; thus, they were not toxicologically significant. In addition, the changes in organ weight were considered to be associated with weight loss.

Contrary to our results, a recent study has shown an increase in serum ALT and AST levels and hepatocellular necrosis after administration of *P. lobata* extract (500 mg/kg/day) for 4 weeks in female mice, suggesting that the main compound puerarin may be responsible for this effect [23]. However, most evidence suggests that *P. lobata* and puerarin have hepatoprotective effects in animal models of hepatotoxicity [35–40]. Also, administration of *P. lobata* extract for 12 weeks did not affect ALT and AST levels in human subjects with elevated serum γ-glutamyl transferase levels due to alcohol consumption [41]. Baicalin, the major compound of *S. baicalensis*, has been reported to induce kidney injury and fibrosis in rats at high doses (≥ 400 mg/kg/day *per os*); yet the highest administered baicalin dose in this study was approximately 200 mg/kg/day, which is lower than the reported toxic dose [24]. Under the experimental conditions of this study, the oral NOAEL of HT047 was found to be greater than 5000 mg/kg/day in rats of both sexes.

Limited data are available on the potential toxicity of *P. lobata*, *S. baicalensis*, and their combination. Chang et al. reported that single oral administration of the polysaccharide fraction of *P. lobata* at doses of up to 5000 mg/kg did not cause any toxicological changes in male and female rats [16]. The oral 50% lethal dose (LD_50_) and ALD of *P. lobata* and *S. baicalensis* water extracts were both greater than 2000 mg/kg in male and female mice [15, 17]. In the acute toxicity studies of Seong et al. [15] and Lee et al. [17] a dose of 2000 mg/kg was set as the highest dose according to the guidelines of the Korean MFDS and OECD. However, a rat dose of 2000 mg/kg does not provide a 10-fold safety margin of the proposed human dose of HT047 (2250 mg/day) and thus a dose of 5000 mg/kg was chosen for this acute toxicity study. Our results of the acute and subchronic toxicity studies, together with previous findings, support the positive safety profile of *P. lobata*, *S. baicalensis*, and their combination. The single and repeated administration of HT047 did not cause any toxic effects in rats at doses of up to 5000 mg/kg/day, which corresponds to approximately 22 times the proposed dose administered in the clinical trial. Thus, it could be presumed that HT047 has a sufficient safety margin.

## Conclusions

In conclusion, the oral ALD and NOAEL of HT047 were > 5000 mg/kg and > 5000 mg/kg/day, respectively, in rats, and no target organs were identified. The present study supports the safety of HT047 as an agent for the treatment of ischemic stroke and further confirms the safety of the clinical use of *P. lobata* and *S. baicalensis* in combination.

## Data Availability

The datasets used and/or analyzed during the current study are available from the corresponding author on reasonable request.

## Abbreviations

A/G: Albumin/globulin
ALD: Approximate lethal dose
ALP: Alkaline phosphatase
ALT: Alanine aminotransferase
AST: Aspartate aminotransferase
BUN: Blood urea nitrogen
HPLC: High-performance liquid chromatography
LD50: 50% lethal dose
MFDS: Ministry of Food and Drug Safety
NOAEL: No-observed-adverse-effect level
OECD: Organization for Economic Co-operation and Development
